# Type I Diabetes—A Rare Adverse Event Described in Patients Receiving Immunotherapy Versus a Side Effect from SARS-CoV-2 Infection

**DOI:** 10.3390/reports8010031

**Published:** 2025-03-14

**Authors:** Raluca-Ileana Pătru, Miruna Ghigeanu, Maria-Alexandra Barbu, Andreea Iuliana Ionescu, Antone-Iordache Ionuț-Lucian

**Affiliations:** 1 Department of Medical Oncology, Colțea Clinical Hospital, 030167 Bucharest, Romania; ileana-raluca.patru@drd.umfcd.ro (R.-I.P.); miruna.ghigeanu@rez.umfcd.ro (M.G.); 2 Department of Oncology, Carol Davila University of Medicine and Pharmacy, 020021 Bucharest, Romania; maria.barbu@umfcd.ro; 3 MedEuropa Center of Oncology and Radiation Therapy, Dobroesti 20a, 022343 Bucharest, Romania; 4 Department of Oncological Radiotherapy and Medical Imaging, Carol Davila University of Medicine and Pharmacy, 020021 Bucharest, Romania; antoneiordachelucian@stud.umfcd.ro

**Keywords:** lung cancer, immunotherapy, type I diabetes, adverse effect, SARS-CoV-2 pandemic

## Abstract

**Background and Clinical Significance**: Lung cancer, a leading cause of global cancer diagnoses, maintains the highest mortality risk despite advances in treatment. Immunotherapy agents, such as anti-programmed death-1/programmed death ligand-1 (PD-1/PD-L1), have revolutionized care for non-small cell lung cancer (NSCLC). However, the success is tempered by the emergence of immune-mediated adverse reactions, including the rare onset of type I diabetes. The incidence of diabetes mellitus increased during the SARS-CoV-2 pandemic. While there are several cases of new-onset diabetes after COVID-19 and COVID-19 vaccination, no case of new-onset type 1 diabetes after COVID-19 was described in an immune checkpoint inhibitor (ICI)-treated patient. **Case Presentation**: A 57-year-old male with stage IV NSCLC (brain and liver metastases) who had been treated with nivolumab for 4 years appeared positive for SARS-CoV-2 infection at a routine check. After two weeks, he was admitted to our clinic with severe fatigue, hyperglycemia, hyponatremia, and hyperkalemia. HbA1c level was normal and serum peptide C was undetectable. Nivolumab treatment was ceased, and the patient became fully dependent on basal–bolus insulin. After 3 months, the patient showed a complete imagistic remission. **Conclusions**: The case presented significant challenges due to the unclear etiology of newly onset diabetes and the uncommon age at which type 1 diabetes is developed. The outcome suggests that anti-PD-1 and SARS-CoV-2 infection can act synergistically.

## 1. Introduction and Clinical Significance

Lung cancer is one of the most common cancers diagnosed worldwide, and while its incidence has been surpassed in women by breast cancer, it still carries the highest cancer mortality risk [1]. Continuous drug development and extensive clinical trials led to a new standard of care in treating these patients, with targeted and immunotherapy agents such as anti-programmed cell death-1 receptor 1/ligand anti (PD-1/PD-L1). While the overall survival in patients diagnosed with locally advanced or metastatic non-small cell lung cancer (mNSCLC) had increased dramatically, an emergence of novel spectrum immune-mediated adverse reactions has been reported by oncologists [2]. The documentation of these reactions poses a significant challenge for clinicians due to their unpredictable nature and inherent difficulty in distinguishing them from patient comorbidities. Notably, the onset of type I diabetes in individuals undergoing immune checkpoint inhibitor (ICI) therapy represents such an event, emphasizing the complexity associated with managing these immunotherapy-related adverse reactions [3]. The relevance and intricacy of these specific adverse reactions were further investigated during the COVID-19 pandemic. While the mortality of SARS-CoV-2 infection was not increased in immunotherapy-treated patients, the risk for severe disease was higher [4]. SARS-CoV-2 infection itself has been linked to long-term effects such as fatigue, headaches, persistent cough, and notably, the occurrence of type I diabetes [5]. Within the context of this paper, we present a compelling case involving a patient diagnosed with stage IV NSCLC undergoing treatment with Nivolumab, who subsequently developed type I diabetes following SARS-CoV-2 infection.

## 2. Case Presentation

**Figure 1:**
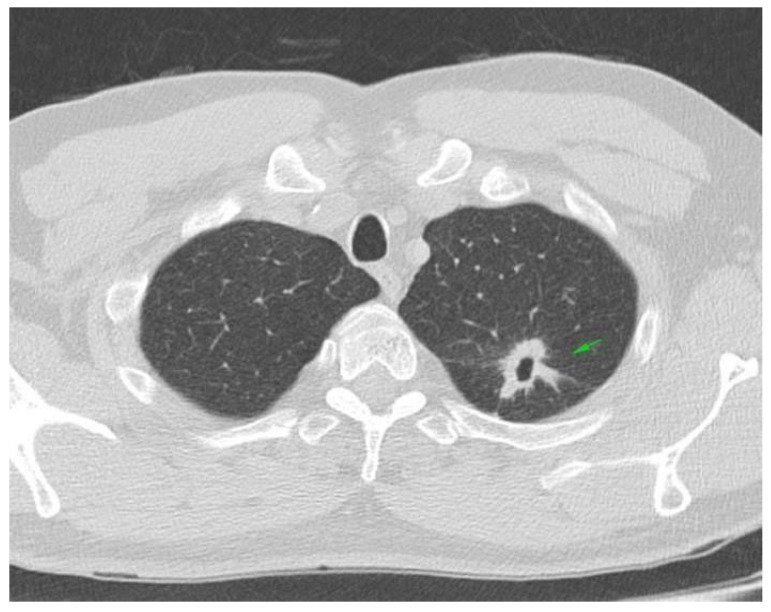
Chest CT scan: left superior lung lobe tumor cT2cN0.

A 57-year-old Caucasian male, heavy smoker, with no familial history of neoplasia or autoimmune disease, presented in March 2017 to the emergency department with persistent cough, night fever, and weight loss. A chest, abdominal, and pelvic CT scan revealed a 40/80 mm tumoral mass in the superior left lobe of the lung, corresponding to cT2 cN0 cM0 stage II lung tumor (Figure 1).

**Figure A1:**
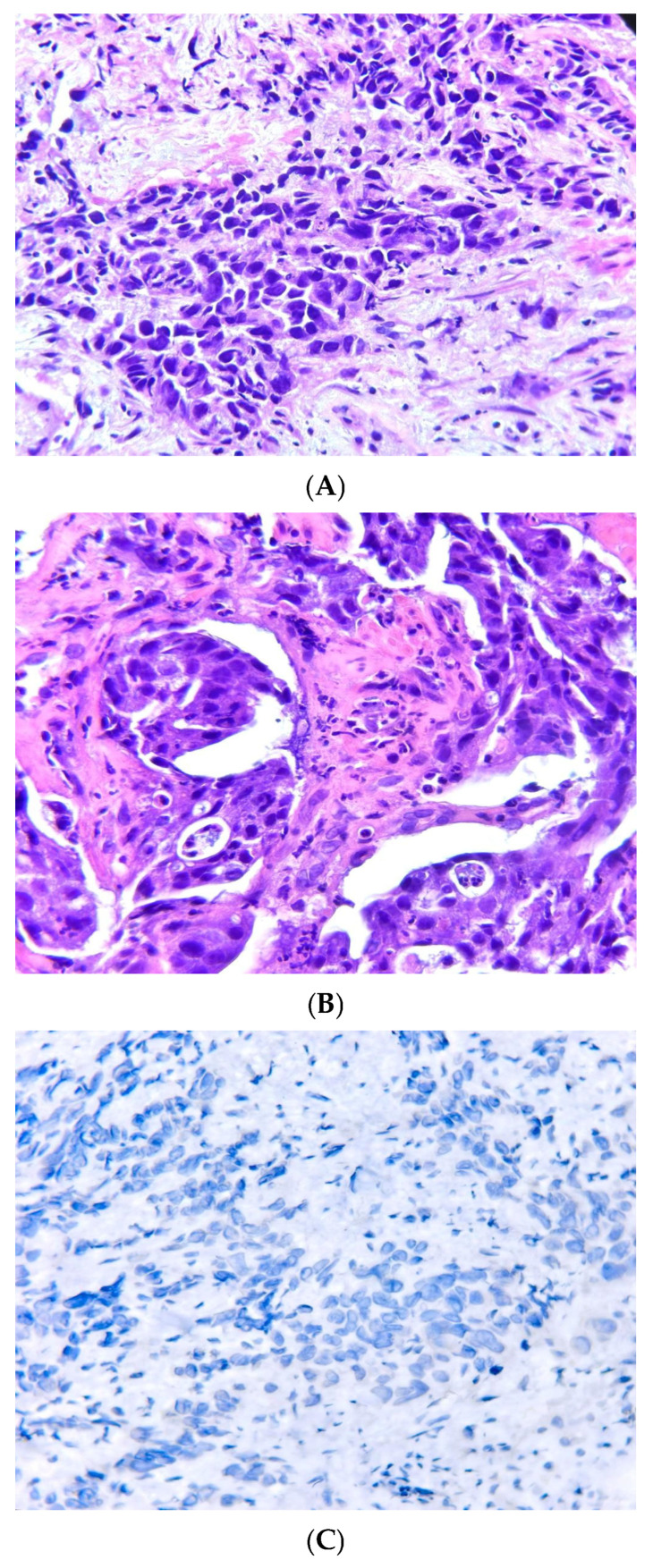
(**A**) HE×200: Invasive lung adenocarcinoma-G3, solid predominant pattern: sheets of neoplastic cells with poorly formed glands. (**B**) HE×200: Invasive lung adenocarcinoma-G2, acinar pattern: variably sized and shaped glands in a fibrotic stroma. (**C**) IHC×400: Invasive lung adenocarcinoma negative for ALK (D5F3 clone Ventana).

The biopsy report identified pulmonary adenocarcinoma (Figure A1). After MDT discussion, the mass was resected, and the pathology report, immunohistochemistry, and genetic testing confirmed the presence of a pulmonary adenocarcinoma EGFR, ALK negative (Figure A1C).

**Figure 2:**
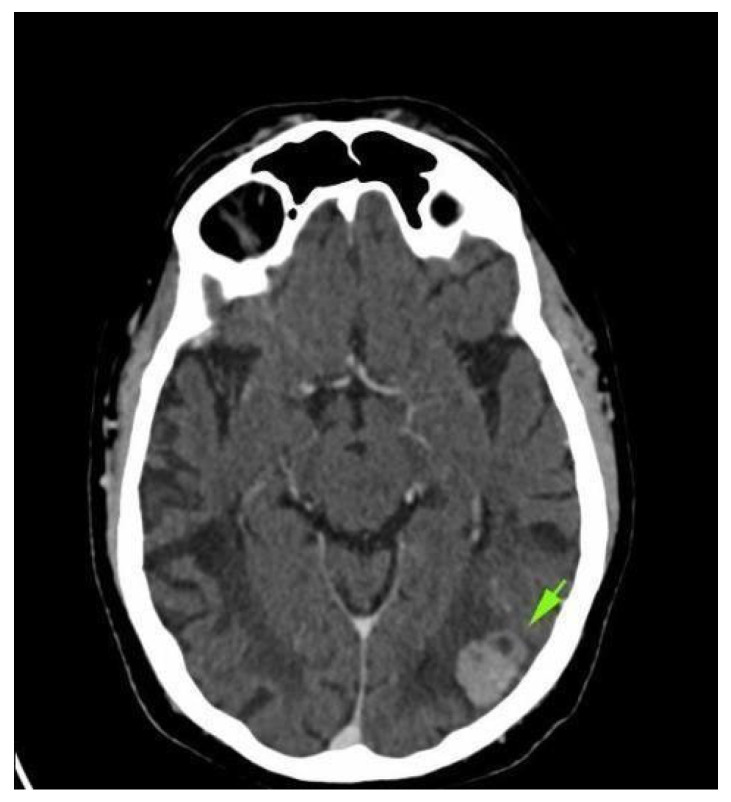
Brain CT scan: left occipital lobe metastases.

**Figure 3:**
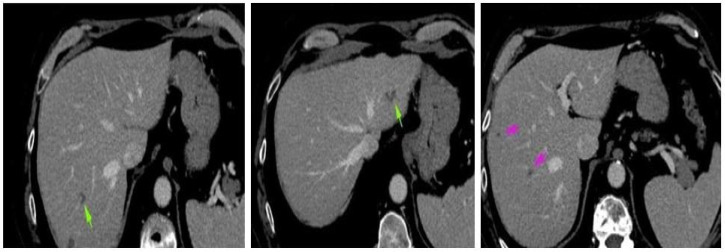
Abdominal CT scan: hepatic metastases.

Four weeks after the intervention, the patient complained of blurred vision, headache, and ataxia. The cerebral CT scan showed two lesions: one in the cerebellum (7/7 mm) and another in the occipital lobe (19/9 mm). The multidisciplinary team confirmed that the patient should start whole brain radiation therapy (WBRT) and subsequent treatment for stage IV lung adenocarcinoma. After finishing WBRT, the patient began therapy with pemetrexed—cisplatin q3w for six cycles, which were very well tolerated. He continued pemetrexed maintenance until June 2018, when his CT scan revealed hepatic and left occipital lobe metastases (Figure 2 and Figure 3). The brain metastases were resected, and the patient received immunotherapy—nivolumab 240 mg q2w.

He continued the treatment for almost four years, with no evidence of oncological disease progression and a very good performance status (ECOG 0) until March 2022, when he was diagnosed with SARS-CoV-2 infection (screening, patient was asymptomatic). After one week of monitoring for signs and symptoms of SARS-CoV-2 infection, the patient returned to the Oncological Department to continue immunotherapy. The blood tests were within parameters, and he received the scheduled dose of Nivolumab.

One week after the scheduled treatment, the patient complained of severe fatigue. Blood tests showed elevated transaminase levels 1.5 times the upper limit normal (AST = 46 U/L, ALT = 97 U/L) and severe hyperglycemia (585 mg/dL), hyponatremia (Na = 130.5 mmol/L), hyperkalemia (6.41 mmol/L), and hypochloremia (89.6 mmol/L); HbA1c was maintained within the normal range, and serum-C peptide was undetectable. Further testing revealed no positive anti-islet cell antibodies or glutamic acid decarboxylase antibodies.

**Figure 4:**
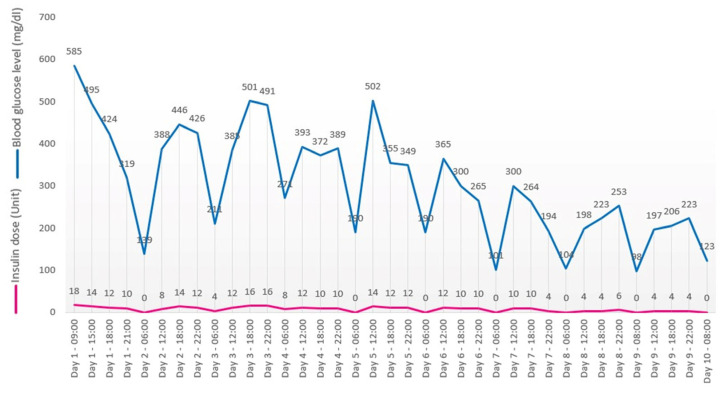
Glucose levels evolution.

The patient was admitted to the medical oncology clinic and received regular intravenous insulin administration under the supervision of a diabetologist. Blood glucose monitoring was performed four times daily, and the insulin dose was prescribed according to the diabetology consultant’s indications (Figure 4). Other risk factors for developing type I diabetes (excepting Nivolumab treatment and SARS-CoV-2 infection) were ruled out: no former history of autoimmune diseases, no familial history of autoimmune diseases, and no enteroviral infection [6]. Unfortunately, genetic testing was not performed.

At the same time, the patient received hypertonic sodium chloride solutions, and the blood test levels were repeated daily. On treatment day ten, the transaminase levels remained high (AST = 58 U/L, ALT = 106 U/L), and blood tests revealed hyperglycemia (123 mg/dL), with normal natremia (Na = 148 mmol/L), kalemia (5.4 mmol/L), and chloremia (99 mmol/L). TSH and fT4 levels were within normal limits.

Any viral or toxic etiology for the high level of transaminases (acetaminophen, dietary supplement, or alcohol use) was ruled out. AST and ALT were under 5 times the upper limit of normal (ULN), with normal bilirubin levels. Therefore, liver enzymes/liver function tests (LFTs) were monitored every 3–5 days. Corticotherapy was not taken into consideration due to very high glucose levels.

After twelve days, the patient was discharged with a prescription for basal–bolus insulin therapy. Nivolumab therapy was permanently discontinued.

**Figure A2:**
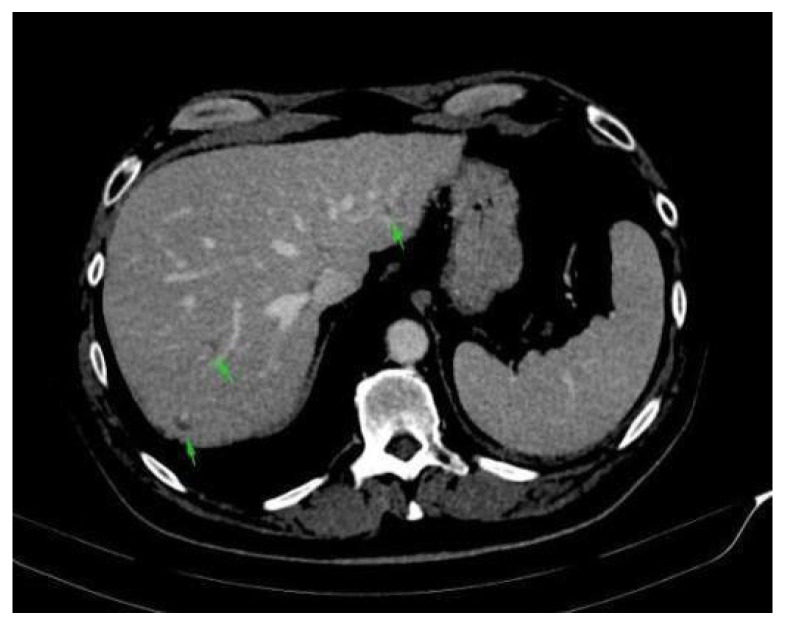
Abdominal CT scan: complete remission of hepatic metastases.

Imaging performed in May 2022—brain MRI, abdominal, and thoracic CT showed complete remission of the oncological disease (Figure A2). The pancreas showed no signs of inflammation. The levels of the transaminases were slightly decreased, and blood glucose levels were within normal limits under prescribed treatment. Serum amylase and lipase were tested, amylase showing a slight increase (121 U/L), while lipase level was normal (193 U/L).

**Figure 5:**
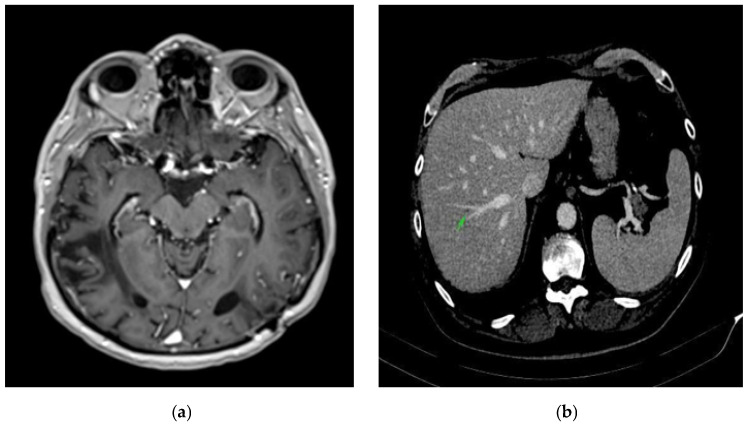
Complete remission. (**a**) Brain CT scan: complete remission of occipital lobe metastases. (**b**) Abdominal CT scan: complete remission of hepatic metastases.

A whole-body CT scan performed in July 2022 (Figure 5a,b) confirmed the complete remission. In November 2022, the brain MRI and the thoracic and abdominal CT scans were within normal limits. The transaminase levels were still 2 times ULN, and the blood glucose levels remained normal.

**Figure 6:**
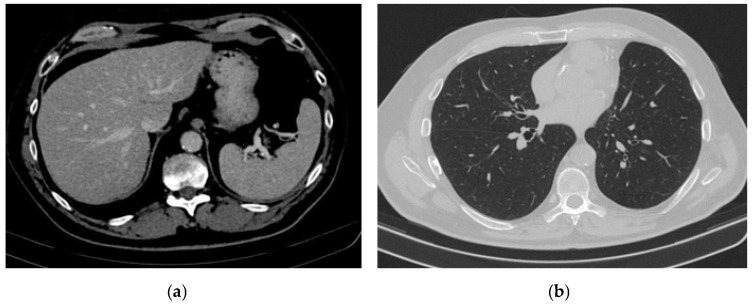
Complete remission. (**a**) Abdominal CT scan: complete remission of hepatic metastases (**b**) Chest CT scan: complete remission.

Periodic CT and MRI scans performed in April 2023 and October 2023 (Figure 6a,b) have revealed no evidence of oncologic disease. To date, the patient has an excellent performance status (ECOG 0), transaminase levels maintained within normal ranges, and diabetes is well controlled with basal–bolus insulin therapy.

## 3. Discussion

Since 2015, immune checkpoint inhibitors have become part of the standard treatment of NSCLC. Together with new treatment advances, a new class of adverse events emerged, and these patients are often diagnosed with endocrinopathies [7,8,9]. Thyroid, pituitary, or adrenal gland toxicities are commonly reported. Diabetes mellitus was reported in 0.2% of cases of patients treated with checkpoint inhibitors in a systematic review that included 7552 patients [5,10,11,12,13]. In real-world data reports, the occurrence of this immunological toxicity appears to be higher than that reported in clinical trials in 2019. V. Tsang published a retrospective study of 538 patients treated with anti-PD-1 immunotherapy for melanoma between 2015 and 2018, and 1.9% of them developed type I diabetes as an adverse event [14]. Additionally, Abdullah et al. present in their review several studies on the onset of diabetes mellitus after nivolumab. Moreover, they emphasize that an elevated number of patients developed a fulminant form of diabetes with acute diabetic ketoacidosis [15]. Several case reports continue to expose the rapid onset of not only type 2 diabetes but type 1 as well. In their work, clinical and paraclinical findings supported the criteria for fulminant type 1 diabetes [16,17,18]. Recently, Pen et al. displayed in their systematic review of 90 patients that early diagnosis of diabetes mellitus was observed in all therapeutic regimens. Moreover, diabetic ketoacidosis was observed in 71% of the patients, while antibodies against islets have been identified in 53%. Notably, nivolumab has been used in five patients as a primary therapy or prior to anti-CTLA-4 [19].

There are a few mechanisms of ICI-induced type I diabetes reported in the literature. The most accepted explanation of the autoimmune process involves the destruction of insulin-secreting β-cells. Altered PD-1/PD-L1 interactions are involved in type 1 diabetes pathogenesis: while PD-L1 expression on β-cells can be increased due to upregulation, lower PD-1 expression on CD4+ T lymphocytes increases islet infiltration with autoreactive CD4+ and CD8+ lymphocytes [20,21]. Pharmacologically blocking the PD-1/PD-L1 pathway is theorized to act on two fronts: islet T lymphocytes release more interferon and activate monocytes that target β-cells, and, in already susceptible patients, due to the alterations detailed in the previous phrase, ICI treatment may be the definitive trigger that kickstarts the type 1 diabetes [21,22].

Against the background of the evolving SARS-CoV-2 pandemic, an observed escalation in the risk of type I diabetes development among infected individuals has been documented, Debuysschere et al. citing numerous meta-analyses and retrospective studies in their review [23]. Additionally, a meta-analysis by Ssentongo et al. compiling data from 47 million patients showed a 66% higher risk for incident diabetes in general, while a meta-analysis by Zhou et al. assessing 60 million patients showed an increase in newly diagnosed type 1 diabetes (HR = 1.46; 95% CI: 1.38–1.55) after COVID-19 infection [24,25].

Steenblock et al. identified SARS-CoV-2 viral RNA in β-cells in six out of eleven human autopsies and demonstrated ACE2 and TMPRSS expression on islet cells [26]. Muller et al. found ACE2 and TMPRSS expressions as well on donor-extracted pancreatic islet cells and demonstrated SARS-CoV-2 replication in infected islet cells. Analyzing the transcriptome, interferon-stimulated genes were upregulated, while genes associated with diabetes were downregulated [27]. Several other pathophysiological links between the SARS-CoV-2 infection and diabetes were discovered in both ex vivo human islet cultures and autopsy samples, ranging from the expression of SARS-CoV N protein in pancreatic tissue from patients with COVID-19 to a decreased number of insulin granules in infected human islet cells. SARS-CoV-2 alone can be an important catalyst for newly discovered diabetes by destroying (through infection) Β-cells and impairing their ability to produce insulin [23]. The COVID-19 cytokine storm and increased interferon levels could also promote autoimmunity to pancreatic islet cells [23,27].

While we presented how immunotherapy and SARS-CoV-2 infection each increase the risk of diabetes, these two factors may act in a synergistic manner. There is currently limited research specifically on mechanisms of diabetes in patients who are infected with COVID-19 and treated with ICI. However, several observational studies and case reports seem to associate SARS-CoV-2 infection with an increased risk of severe immune-related adverse events (irAEs), especially pneumonitis [28,29,30]. The proposed mechanism of such synergy is characterized by dysregulated immunity: in some cases, SARS-CoV-2 infection determines cytokine release syndrome (CRS), which can also be caused by ICI treatment. Both instances of CRS can be treated with tocilizumab [28,31]. There is currently conflicting evidence regarding an association between severe COVID-19 and ICI treatment, with some studies reporting a higher rate of hospitalization for SARS-CoV-2 infection in ICI-treated patients [31].

The synergistic action of immunotherapy and SARS-CoV-2 infection is further underlined by the late onset of type 1 diabetes in our patient, compared to the median time for developing this irAE: 7 to 25 weeks, depending on the study [21]. This might suggest that SARS-CoV-2 acted as a trigger for type 1 diabetes onset in a patient who had been treated with nivolumab for 4 years.

In clinical practice, the emergence of type I diabetes in patients undergoing treatment with immune checkpoint inhibitors introduces clinical dilemmas. Recent epidemiological data suggests that a significant proportion of type I diabetes cases manifest in adults, frequently misclassified as type II diabetes [32]. The age of our patient (57) would more probably indicate type II diabetes in a clinical setting. However, there are other cases of newly onset type I diabetes after SARS-CoV-2 infection/vaccination that present having a similar age of around 50–55 years [33,34,35]. A systematic review of type 1 diabetes after COVID vaccination case reports implies a median age of 50.5 years [36]. Chen et al. report in a cohort study of 30,000 ICI-treated patients that the mean age for patients that developed type 1 diabetes was 68, far higher than the expected age of onset for this disease [37].

Regarding the complete remission of our patient, there are several cases of cancer remission after SARS-CoV-2 infection in renal, colonic, and biliary carcinomas from patients treated with either chemotherapy or ICI, although the mechanism that results in such outcomes is not completely clear [38,39]. Two hypotheses are discussed by Meo et al.: a priming hypothesis involving the activation of the immune system by inflammatory factors and an oncolytic hypothesis where SARS-CoV-2 acts as an oncolytic virus that infects the tumoral cells. The released pathogen-associated molecular patterns and damage-associated molecular patterns in return create tumor-specific T-cells, leading to an abscopal effect [38]. Consequently, irAEs are correlated with increased rates of objective response, overall survival, and progression-free survival in patients with NSCLC [4,40]. Although the mechanisms of this effect are unclear, it might be speculated that mimicry between host antigens and cancer antigens is a primary cause [4].

As we showed in our case presentation, the evolution of type I diabetes can be fulminant. The management of this immunotherapy-related toxicity depends on the degree of hyperglycemia. In patients with mildly elevated blood glucose levels, the oncological treatment can be administered safely under close monitoring. If the patient presents with severe hyperglycemia and DKA, the oncological treatment must be stopped, and insulin therapy is mandatory. ICI-related diabetes is irreversible, and the patients require life-long insulin treatment [41,42].

## 4. Conclusions

Anti-PD-1 drug-induced type I diabetes constitutes a notable and severe toxicity associated with immunotherapy. The incidence of this endocrinopathy is potentially underestimated in clinical studies. The emergence of this endocrinopathy in a patient with cancer undergoing immunotherapy presents a substantial clinical challenge, given the complex nature of its etiology: ICI-induced, COVID-19-induced, or a synergistic effect.

We consider that this case report underpins a unique case where the association of immunotherapy and SARS-CoV-2 results in a rare irAE. Even if our patient had been treated with nivolumab for over 4 years and did not develop type 1 diabetes mellitus, at two weeks after an asymptomatic infection with SARS-CoV-2, he suffered a fulminant manifestation of this disease.

The complete imagistic remission of oncologic disease shows how the same factors involved in a severe irAE could benefit the patient if the immune system is stimulated and properly directed toward the tumor. Unfortunately, there is no rigorous scientific evidence to support this case.

Further research is necessary in order to understand the precise mechanism of ICI-induced type I diabetes.

This case report adds valuable insights into the challenges of managing immunotherapy-related complications, emphasizing the need for vigilance, multidisciplinary collaboration, and continuous monitoring to optimize patient outcomes.

## Data Availability

The dataset generated is available from the corresponding author upon reasonable request. The data are not publicly available due to privacy concerns.
